# Characteristics of chronic obstructive pulmonary disease patients with robust progression of emphysematous change

**DOI:** 10.1038/s41598-021-87724-8

**Published:** 2021-05-05

**Authors:** Akihiro Tsutsumi, Shotaro Chubachi, Hidehiro Irie, Mamoru Sasaki, Yoshitake Yamada, Hiroaki Sugiura, Masahiro Jinzaki, Hidetoshi Nakamura, Koichiro Asano, Tomoko Betsuyaku, Koichi Fukunaga

**Affiliations:** 1 Division of Pulmonary Medicine, Department of Medicine, Keio University School of Medicine, 35 Shinanomachi, Shinjuku-ku, Tokyo, 160-8582 Japan; 2 Department of Radiology, Keio University School of Medicine, 35 Shinanomachi, Shinjuku-ku, Tokyo, 160-8582 Japan; 3 Division of Pulmonary Medicine, Saitama Medical University Hospital, 38 Morohongo Moroyama-machi, Iruma-gun, Saitama, 350-0495 Japan; 4 Division of Pulmonary Medicine, Department of Medicine, Tokai University School of Medicine, 143 Shimokasuya, Isehara-shi, Kanagawa, 259-1193 Japan

**Keywords:** Epidemiology, Outcomes research, Translational research

## Abstract

Emphysema is a major pathological change in chronic obstructive pulmonary disease (COPD). However, the annual changes in the progression of emphysematous have not been investigated. We aimed to determine possible baseline predicting factors of the change in emphysematous progression in a subgroup of COPD patients who demonstrated rapid progression. In this observational study, we analyzed patients with COPD who were followed up by computed tomography (CT) at least two times over a 3-year period (n = 217). We divided the annual change in the low attenuation area percentage (LAA%) into quartiles and defined a rapid progression group (n = 54) and a non-progression group (n = 163). Predictors of future changes in emphysematous progression differed from predictors of high LAA% at baseline. On multivariate logistic regression analysis, low blood eosinophilic count (odds ratio [OR], 3.22; *P* = 0.04) and having osteoporosis (OR, 2.13; *P* = 0.03) were related to rapid changes in emphysematous progression. There was no difference in baseline nutritional parameters, but nutritional parameters deteriorated in parallel with changes in emphysematous progression. Herein, we clarified the predictors of changes in emphysematous progression and concomitant deterioration of nutritional status in COPD patients.

## Introduction

Chronic obstructive pulmonary disease (COPD) is characterized by persistent respiratory symptoms and airflow limitation^1^. Emphysema is a major pathological change of COPD that is characterized by abnormal and permanent enlargement of distal airspaces as well as by alveolar wall destruction^2^. Airflow limitation is the main characteristic of COPD, but the severity of emphysema differs significantly among individuals who have similar forced expiratory volume in 1 s (FEV_1_)^3^. Chest computed tomography (CT) has been the most accurate and minimally invasive technique used for the diagnosis of emphysema^4^, and CT-diagnosed emphysema is strongly associated with more rapid decline in FEV_1_^5^, worse health status^6^, and increased mortality rates^7^.

Progression of emphysema was found to be more sensitive using chest CT than by using lung function parameters^8^ and reported the utility as treatment outcome in COPD patients^9^. It has been reported that the progression of emphysema, as well as pulmonary function decline, varies between patients^10^. Thus, factors that could predict emphysematous progression are required. A recent study showed that sex, smoking status, plasma levels of surfactant protein D (SP-D), soluble receptor for advanced glycation endproducts (sRAGE)^10^, and the leptin/adiponectin ratio^11^ were associated with changes in emphysematous progression. Several recent large-scale cohort studies evaluated the changes in emphysematous progression; however subjects underwent chest CT at baseline and 3- to 5-year follow-up in these studies^10,12^. Emphysema quantification is very sensitive to various conditions, including the level of inspiration. Thus, when assessing longitudinal changes by chest CT, the appropriate number of times, calibration of different CT scanners, and the scanning protocol used are important^13^. However, the annual changes in emphysematous progression on chest CT have not been assessed in COPD patients.

Systemic manifestations and comorbidities of COPD also contribute to the different clinical phenotypes and alterations in body weight and composition, from cachexia to obesity, demanding specific management^14^. Several previous reports have demonstrated the association among emphysema, low body mass index (BMI), and osteoporosis in COPD patients^15–17^. We hypothesized that low BMI and having osteoporosis could predict future changes in emphysematous progression and that the annual change in emphysema would correlate with the annual change in BMI and bone mineral density (BMD). Thus, the aims of this study were threefold: 1) to identify a subgroup of COPD patients who demonstrate rapid progression of emphysematous change during a 3-year follow-up period; 2) to identify possible baseline factors, including comorbidities, which could predict the rapid progression of emphysematous change; and 3) to assess factors that change synchronously with emphysematous progression in COPD patients.

## Methods

### Study population

The overall design of the Keio COPD Comorbidity Research (K-CCR) has been published previously^15,18^. This study was a 3-year, prospective, observational study that enrolled 572 men and women, aged 40–91 years, diagnosed with COPD (n = 440) or as being at risk of COPD (n = 132) by pulmonary physicians, from April 2010 to December 2012. Data of COPD patients who underwent CT at least two times over a 3-year period (n = 217) were analyzed (Supplemental Fig. 1). All patients were clinically stable at all assessments and had no exacerbations for at least 1-month pre-enrollment.

**Figure 1:**
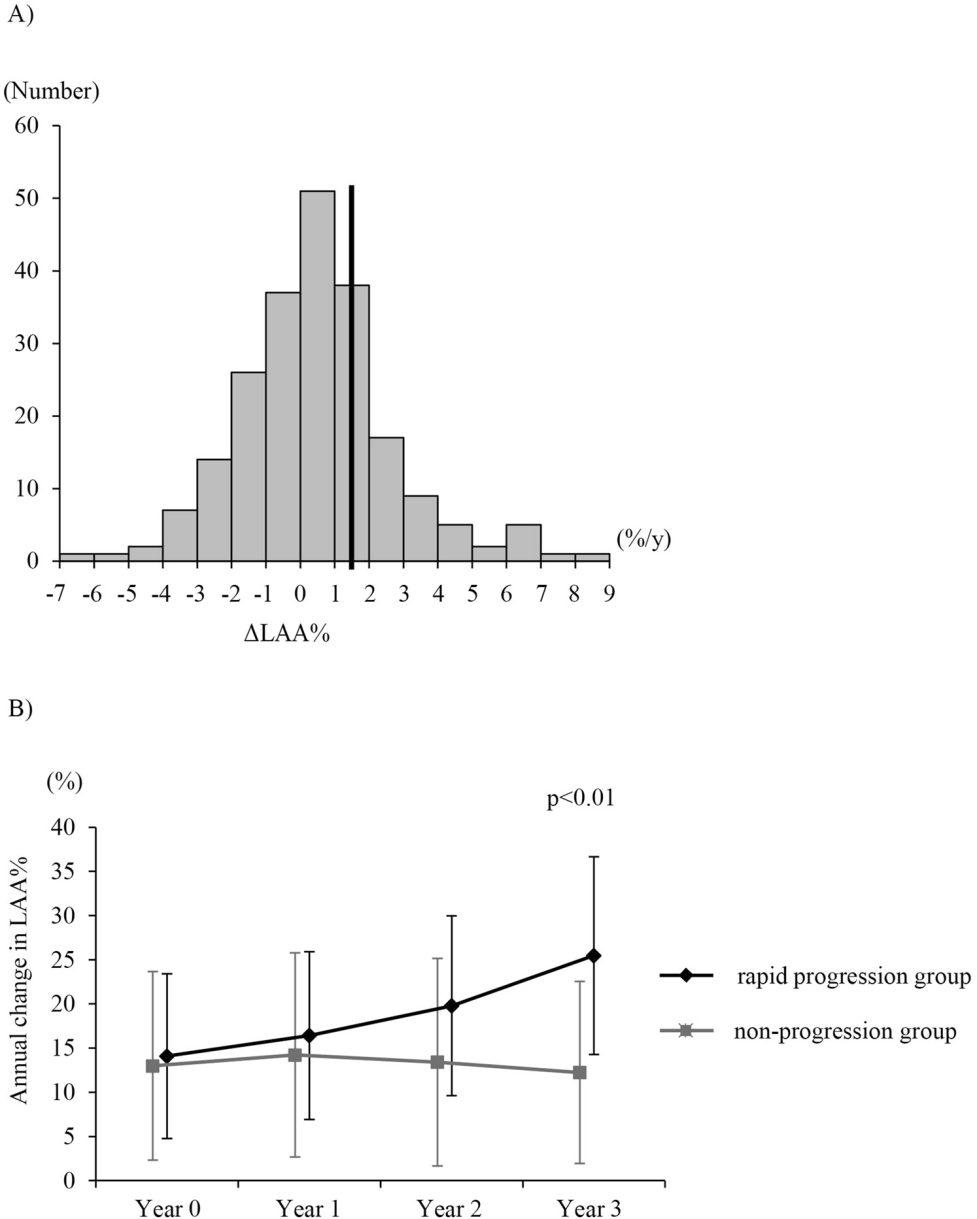
Distribution of annual changes in LAA% and time-dependency over 3 years. (**A**) Distribution of the annual changes in LAA% over the 3-year period. The mean ± SD of ΔLAA%/year was 0.47 ± 2.28. We had arbitrarily defined the cut-off value of ΔLAA%/year, based on the upper quartile value, as 1.48%/year. (**B**) Overall time-dependent LAA% in the rapid progression and non-progression groups. Data are shown as mean ± SD. *LAA* low attenuation area.

Written informed consent for the use of data was obtained from each patient, and the study (University Hospital Medical Information Network; UMIN000003470) was approved by the ethics committees of Keio University and its affiliated hospitals (20,090,008). All methods were performed in accordance with the relevant guidelines and regulations.

### Assessment of clinical parameters

At enrollment and annually, a full medical and smoking history, and current pharmacological treatment information, were obtained^18^. Comorbid conditions were diagnosed based on clinical history and physical examination, supported by medical record review^18,19^. Spirometry was performed in all patients using an electronic spirometer (CHESTAC-9800; CHEST, Tokyo, Japan) according to the American Thoracic Society guidelines^20^. Body mass composition, i.e., fat-free mass (FFM) and muscle mass (MM), was assessed using a Tanita BC-308/BC-309 bioelectrical impedance analyzer (Tanita, Inc., Tokyo, Japan)^21^. The FFM index (FFMI) was calculated as FFM divided by height-squared^22^.

Blood samples were collected at baseline and annually thereafter. A pre-specified eosinophil cut-off of 300 cells/μl was used to determine association with the change in emphysematous progression^23,24^.

The Japanese version of the COPD assessment test (CAT)^25^ and the St. George’s Respiratory Questionnaire (SGRQ)^26–28^ was performed at baseline. Independent investigators retrospectively judged the number and severity of exacerbations based on reviews of physicians’ medical records^29^.

### Assessment of low attenuation areas and airway wall thickness on chest CT

CT was performed using four multi-detector CT scanners, including 64-detector CT (LightSpeed VCT and Discovery CT 750 HD, General Electric Medical Systems, Milwaukee, WI, USA, or Aquilion 64, Toshiba Medical Systems, Otawara, Japan) or 256-detector CT (Revolution CT, General Electric Medical Systems, Milwaukee, WI, USA) scanners. All subjects underwent volumetric CT at full inspiration and at the end of a normal expiration. Scanning parameters for each scanner were as follows: the detector collimation was 0.5–0.625 mm; beam pitch, 0.813–0.984; reconstruction thickness, 1.0–1.25 mm; reconstruction interval, 1.0–1.5 mm; rotation time, 0.35–0.5 s; tube voltage, 120 kVp; tube current, Auto mAs (standard deviation [SD] = 12–15); and reconstruction kernel, chest for GE machine or FC 50 for Toshiba machine. For calibration among four CT scanners, a test object (Multipurpose Chest Phantom N1; Kyoto Kagaku, Kyoto, Japan) was scanned at the start of the study using each scanner^15^. (Supplemental Fig. 2). The emphysema extent was quantified as the ratio of the low attenuation area to the total lung volume (LAA%), with Hounsfield units < − 950 (AZE Ltd., Tokyo, Japan)^15^.

**Figure 2:**
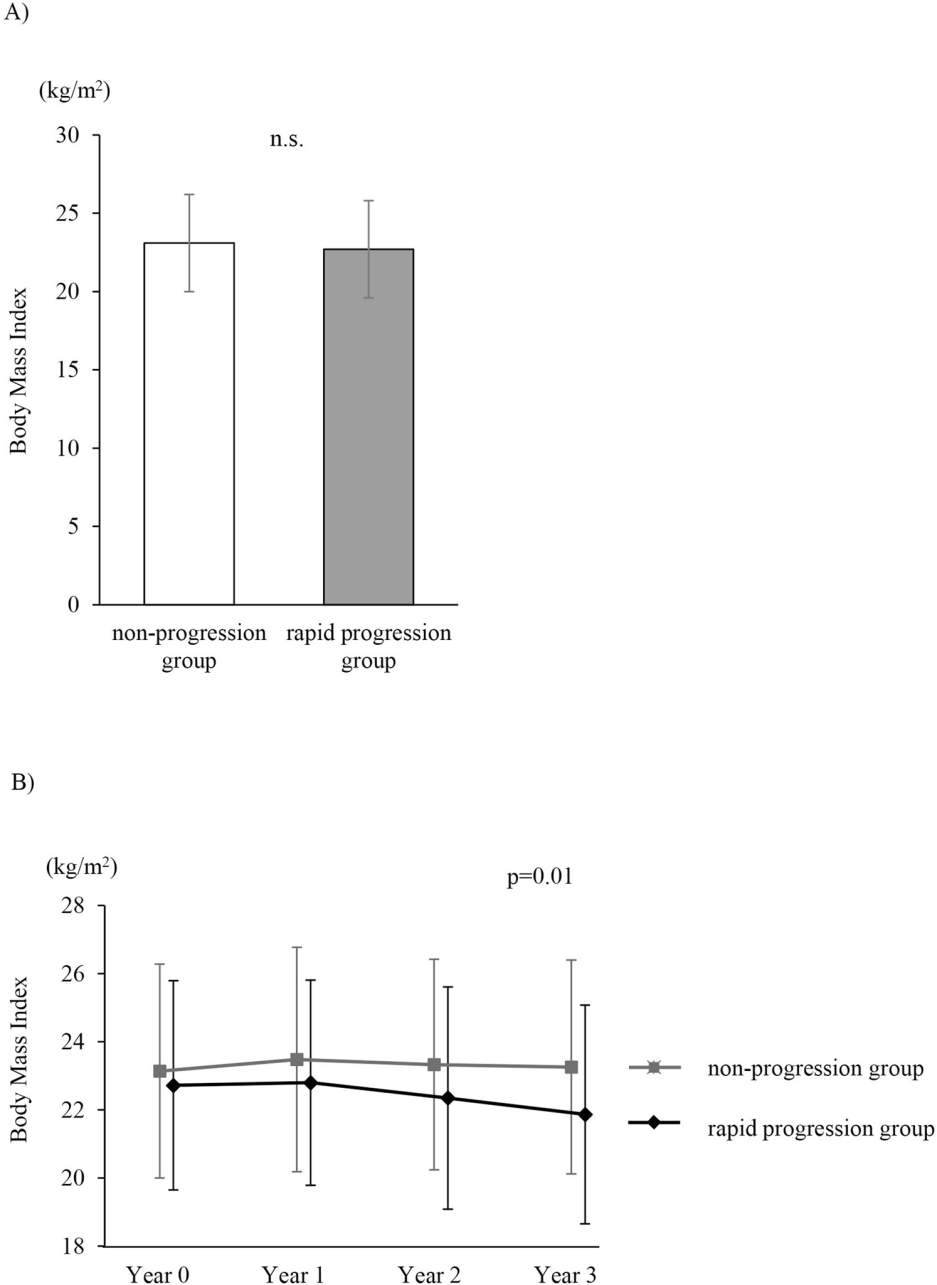
Comparison of BMI between the rapid progression group and non-progression group. (**A**) BMI at baseline. (**B**) Annual change in BMI over 3 years of follow-up. Data are shown as mean ± SD. *BMI* body mass index.

As shown in Supplemental Fig. 2A below, the phantom was first scanned on one control CT scanner. The LAA% of this phantom varies depending on the cutoff HU value. When the cutoff LAA value was set at − 950 HU on the control CT scanner, LAA% was 76%. The same phantom was scanned on the other four scanners, and the cutoff HU level specific to each model by which LAA% became 76% was determined to allow adjustment (Supplemental Fig. 2B).

### Dual X-ray absorptiometry

Dual X-ray absorptiometry (DXA) measurements of bone mineral density (BMD) were performed at both hips and lumbar spine using a Hologic 4500A Discovery bone densitometer (HOLOGIC, Bedford, MA). Osteoporosis diagnosis was based on the lowest T-score of these locations, according to World Health Organization criteria^30^.

### Statistical analysis

Data were compared between two groups using Student’s *t*- and χ^2^ tests; three groups were compared by analysis of variance and χ^2^ tests. LAA%, BMD, and BMI were compared by percent changes from baseline values. Excel (Microsoft Inc. Redmond, WA) was used to calculate the linear regression through data points, including data in the middle^31^. Univariate and multivariate logistic regression analyses were performed to assess factors affecting change in emphysematous progression. Correlations between continuous variables were evaluated using Pearson’s correlation coefficient. Multivariate logistic regression analysis was performed using related factors that either reached significance or trended towards an association on univariate analyses. The changes of LAA%, BMI or BMD at each visit were estimated by a linear mixed effect model with groups; non-progression and rapid progression groups, time point, and time-by- groups interaction as fixed effects, subject as a random effect, to obtain point estimates and 95% confidence interval. The correlation structure was assumed as compound-symmetry structure. For all tests, two-sided p-values < 0.05 were considered significant. Data were analyzed using JMP 14 software (SAS Institute, Cary, NC).

## Results

### Clinical features of the study populations

Table 1 shows the baseline characteristics of the study participants. The average age of the COPD patients was 72.4 ± 8.4 years. The number of COPD patients in Global Initiative for Chronic Obstructive Lung Disease grades 1, 2, 3, and 4 were 30.8%, 47.0%, 17.5%, and 4.6%, respectively.

**Table 1:** Baseline characteristics of the study population.

	*N* = *217*
Age, year	72.4 ± 8.4
Sex, female, N (%)	19 (8.8)
Smoking index, pack-years	53.5 ± 31.0
Current smoker, N (%)	19 (8.8)
BMI, kg/m^2^	23.0 ± 3.1
FFMI, kg/m^2^	17.7 ± 2.0
FEV_1_, ml	1807.6 ± 630.7
%FEV_1_, %	67.8 ± 21.0
COPD grade*, 1/2/3/4 (%)	67/102/38/10 (30.8/47.0/17.5/4.6)
Bronchodilator (%)	148 (68.2)
ICS, N %	55 (25.3)

Data are presented as mean ± SD or number (%).

*BMI* body mass index, *FFMI* fat-free mass index, *FEV*_*1*_ forced expiratory volume in 1 s, *%FEV*_*1*_ forced expiratory volume in 1 s as a percentage of predicted forced expiratory volume in 1 s, *COPD* chronic obstructive pulmonary disease, *GOLD* Global Initiative for Chronic Obstructive Lung Disease, *ICS* inhaled corticosteroids.

*Defined by the Global Initiative for Chronic Obstructive Lung Disease.

### Distribution of annual changes in LAA% over a 3-year period

The annual changes in LAA% (ΔLAA%/year) is shown in Fig. 1A. The mean ΔLAA%/year was 0.47. We had arbitrarily defined the cut-off value of ΔLAA%/year based on an upper quartile value of 1.48%/year (Rapid progression group; n = 54, Non-progression group; n = 163). Figure 1B shows the longitudinal change in LAA% over the 3-year period in the two groups. The difference in the rate of LAA% change among the two groups was significant (*P* < 0.01). ΔLAA%/year significantly correlated with ΔLAA volume/year (r = 0.65, *P* < 0.01), but not ΔTotal lung volume (TLV)/year (r = 0.03, *P* = 0.77).

### Baseline characteristics of COPD patients with emphysema and changes in emphysematous progression

The baseline characteristics of COPD patients, stratified by the baseline LAA% and annual change of LAA%, are shown in Table 2 and Supplemental Table 1. Patients with mild (LAA% ≥ 10% to < 20%) and moderate/severe(LAA% ≥ 20%) emphysema had lower lung function, lower BMI, more osteoporosis, and worse quality of life (QOL) scores than those without emphysema (LAA% < 10%) (Supplemental Table 1). In contrast, there were no differences in lung function, BMI, and QOL scores between the rapid progression group and the non-progression group. Additionally, the baseline LAA% and prevalence of interstitial pneumonia did not differ between these two groups (LAA%: *P* = 0.51; prevalence of interstitial pneumonia: *P* = 0.25). Interestingly, the eosinophil count was lower in the rapid emphysema group than in the non-progression group (eosinophil count: 150.7 ± 89.5 cells/mm^3^ vs. 226.9 ± 215.7 cells/mm^3^, *P* = 0.01) (Table 2). These results imply that baseline LAA% does not predict the rate of future changes in emphysematous progression and the related factors differ between baseline advanced emphysema and changes in emphysematous progression.

**Table 2:** Comparison of baseline characteristics according to group.

	Non-progression group	Rapid progression group	*P*-value
	*N* = *163*	*N* = *54*	
Age, years	72.2 ± 8.5	73.0 ± 8.2	0.54
Sex, female, N (%)	11 (6.8)	8 (14.8)	0.07
Smoking index, pack-years	54.1 ± 31.8	51.5 ± 28.9	0.60
Current Smoker, N (%)	14 (8.8)	5 (9.3)	0.92
Lung function			
FEV_1_, ml	1838.7 ± 612.1	1713.7 ± 681.5	0.21
%FEV_1_, %	68.0 ± 19.6	67.3 ± 24.9	0.84
LAA%, %	13.0 ± 10.7	14.1 ± 9.3	0.51
WA%, %	54.3 ± 8.6	56.4 ± 8.6	0.12
Other Pulmonary Disease			
Interstitial Pneumonia, (%)	17 (10.5)	9 (16.4)	0.25
Asthma, (%)	30 (18.4)	11 (20.4)	0.64
Laboratory values			
Blood neutrophil count, cells/mm^3^	3937.5 ± 1479.1	3937.5 ± 1646.6	0.60
Blood eosinophil count, cells/mm^3^	226.9 ± 215.7	150.7 ± 89.5	0.01
SAA, µg/ml	11.7 ± 25.8	27.4 ± 99.7	0.08
CRP, mg/dl	0.26 ± 0.71	0.44 ± 1.41	0.23
Patient-reported outcomes			
CAT score	11.7 ± 7.8	12.4 ± 8.3	0.53
SGRQ total score	25.9 ± 17.9	28.4 ± 18.5	0.42
Bronchodilator (%)	108 (73.0)	40 (72.7)	0.40
ICS, N %	42 (25.9)	13 (23.6)	0.74

Data are presented as mean ± SD or number (%).

*FEV*_*1*_ forced expiratory volume in 1 s, *%FEV*_*1*_ forced expiratory volume in 1 s as a percentage of predicted forced expiratory volume in 1 s, *LAA%* the ratio of low attenuation area to total lung volume, *WA%* the percentage of airway wall area, *SAA* serum amyloid A, *CRP* C-reactive protein, *CAT* chronic obstructive pulmonary disease assessment test, *SGRQ* St. George’s Respiratory Questionnaire, *ICS* inhaled corticosteroids.

### Relationships between nutritional status and changes in emphysematous progression in COPD patients

At baseline, there was no difference in BMI between the rapid progression group and the non-progression group (Fig. 2A). In contrast, follow-up analysis indicated that the difference in the rate of BMI change among the two groups was significant (*P* = 0.01) (Fig. 2B). As well as ΔBMI/year (r = − 0.21, *P* < 0.01), ΔFFMI/year (r = − 0.20, *P* < 0.01) and ΔMuscle mass/year (r = − 0.20, *P* < 0.01) correlated weakly but significantly with ΔLAA%/year. (Table 3).

**Table 3:** Correlation between annual ΔLAA% and nutritional status change.

	*r*	*P*-value
Δ BMI	− 0.21	< 0.01
Δ FFMI	− 0.20	< 0.01
Δ Muscle Mass	− 0.20	< 0.01

*BMI* body mass index, *FFMI* fat-free mass index, *LAA%* the ratio of low attenuation area to total lung volume.

### Relationships between BMD and changes in emphysematous progression in COPD patients

**Figure 3:**
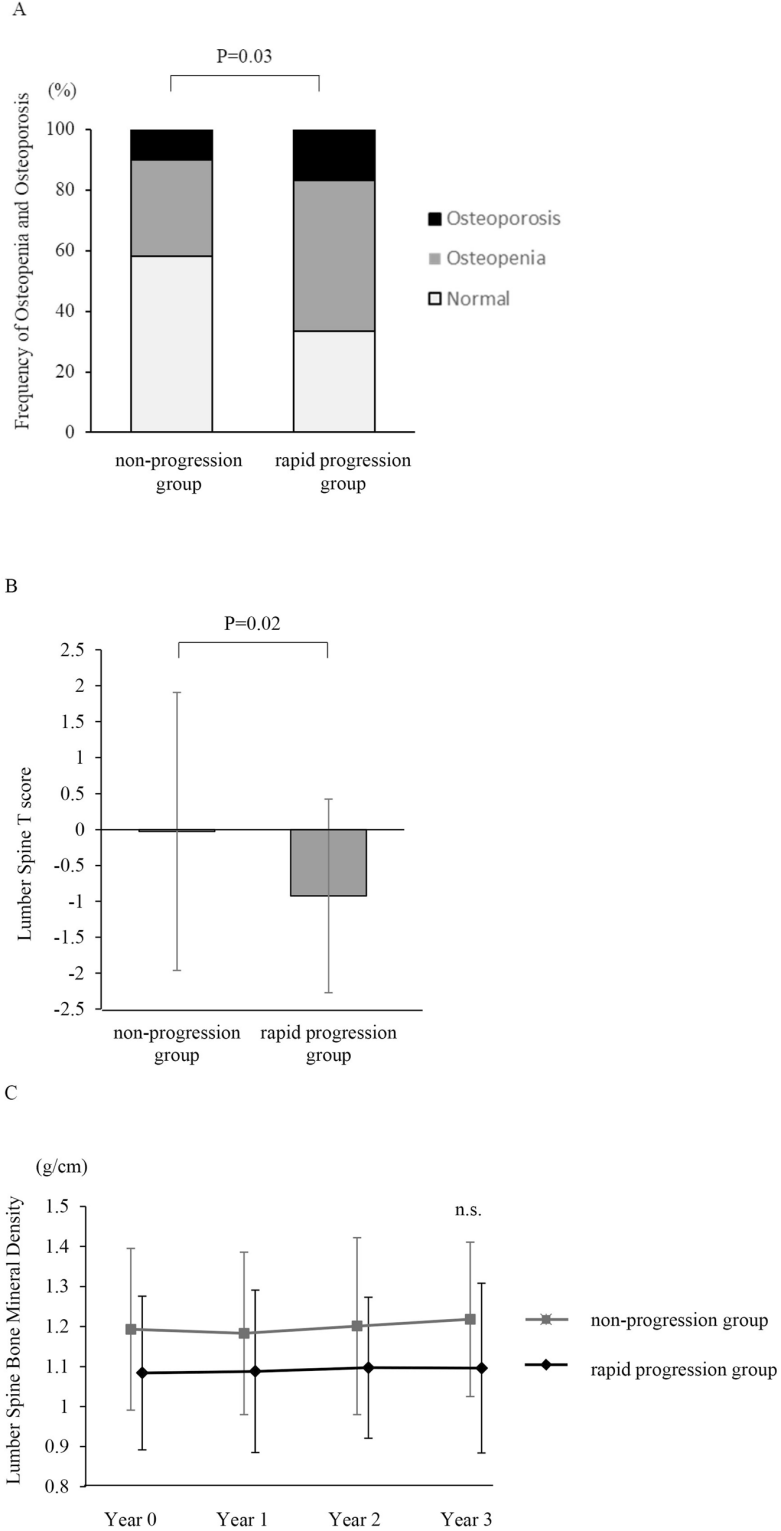
Relationships between lumbar BMD and changes in emphysematous progression in COPD patients. (**A**) Frequency of osteopenia and osteoporosis in the non-progression group and rapid progression group. (**B**) Comparison of baseline T score in the lumbar spine between the two groups. (**C**) Annual change in BMD in the lumbar spine in the two groups over 3 years of follow-up. Data are shown as mean ± SD. *BMD* bone mineral density.

The ratio of patients with osteoporosis and osteopenia was higher in COPD patients in the rapid progression group than in those in the non-progression group (osteoporosis: 22.5% vs. 10.3%; osteopenia: 36.7% vs. 29.5%, *P* = 0.03) (Fig. 3A). Additionally, the baseline BMD at all three parts of the body were significantly lower in the rapid progression group than in the non-progression group (lumbar spine: *P* = 0.03; right femur: *P* = 0.02; left femur: *P* = 0.03) (Fig. 3B, Supplemental Fig. 3A, B). Follow-up analysis over 3 years indicated that the difference in BMD between the two groups was statistically significant (lumbar spine: *P* < 0.01; right femur: *P* < 0.01; left femur: *P* < 0.01), but there was no significant difference in the rate of BMD change between the two groups (lumbar spine: *P* = 0.80; right femur: *P* = 0.88; left femur: *P* = 0.76) (Fig. 3C, Supplemental Fig. 3C, D).

### Predictors and factors showing synchronized progression with emphysematous change in COPD patients

We assessed the predictors of future changes in emphysematous progression using multivariate logistic analysis in which we included several factors that reached significance on univariate analysis (Tables 4). Low blood eosinophilia (< 300 cells/μl) (odds ratio [OR] 3.22, *P* = 0.04), having osteoporosis or osteopenia (OR 2.13, *P* = 0.03) independently predicted future changes in emphysematous progression. There was no difference in the incidence of moderate and severe exacerbations, change in smoking habits, or change in treatment between the rapid emphysema group and the non-progression group over the 3-year period (Supplemental Table 2). Also, there were no differences in the he annual ΔCAT score and ΔSGRQ total score between the two groups (Supplemental Fig. 4).

**Table 4:** Predictors of LAA% rapid progression by univariate and multivariate logistic regression analysis.

Parameters	Univariate analysis Odds Ratio (95% CI)	*P*-value
Age	1.74 (0.29–10.41)	0.54
Sex, female	2.40 (0.91–6.33)	0.08
BMI	0.47 (0.08–2.64)	0.39
FFMI	0.46 (0.07–3.04)	0.42
Pack-year	0.57 (0.08–4.40)	0.59
Current smoker	1.06 (0.36–3.08)	0.92
Osteoporosis or Osteopenia vs normal	2.20 (1.14–4.25)	0.02
%FEV_1_ < 70%	1.06 (0.57–1.97)	0.84
Low Attenuation Area > 10%	1.33 (0.72–2.48)	0.36
Low Attenuation Area > 20%	1.37 (0.70–2.68)	0.37
Blood eosinophil count < 300 cells/mm^3^	2.75 (1.02–7.45)	0.03
Bronchodilator (%)	1.29 (0.65–2.54)	0.46
ICS, N %	0.80 (0.38–1.65)	0.54
Parameters	Multivariate analysis Odds Ratio (95% CI)	*P*-value
Osteoporosis or Osteopenia vs normal	2.13 (1.09–4.14)	0.03
Blood eosinophil count < 300 cells/mm^3^	3.22 (1.07–9.66)	0.04

*BMI* body mass index, *CI* confidence interval, *FFMI* fat-free mass index, *%FEV*_*1*_ forced expiratory volume in 1 s as a percentage of predicted forced expiratory volume in 1 s, *LAA%* the ratio of low attenuation area to total lung volume, *ICS* inhaled corticosteroids.

### Ethics approval and consent to participate

Written informed consent for the use of data was obtained from each patient. This study was registered on the University Hospital Medical Information Network (UMIN000003470) and was approved by the ethics committees of Keio University and its affiliated hospitals (20,090,008).

## Discussion

In this longitudinal study in patients with COPD, we identified possible baseline factors, including comorbidities, that could predict the rapid progression of emphysematous change at three time points; this has not been reported previously. We demonstrated that having osteoporosis and low blood eosinophilia were predictors of future rapid changes in emphysematous progression; additionally, cachexia and health status deteriorated with changes in emphysematous progression.

Previous studies, including our own, have shown an association between emphysema and osteoporosis^15,16^. However, the influence of having osteoporosis on changes in emphysematous progression has remained unclear. The present study showed that having osteoporosis is an important predictor of not only baseline emphysema presence, but also of future changes in emphysematous progression in COPD patients. These results imply that osteoporosis is closely related to emphysema. Systemic inflammation is a plausible mechanistic link between emphysema and osteoporosis^32,33^. However, this concept had not been considered in detail to date. Future studies should focus on the development of targeted therapies designed to prevent the progression of both these disease processes.

Cachexia and muscle wasting are well-recognized comorbidities in COPD patients, and a number of studies have reported that these comorbidities contribute to decreased QOL^31^ and increased mortality^35,36^. In a previous study, baseline BMI and FFMI were not related to baseline LAA%, but ΔBMI, ΔFFMI, and other nutrition indexes were correlated with changes in emphysematous progression. These results were in line with previous studies that demonstrated that lung volume reduction surgery (LVRS) significantly improved nutritional status^37^. Additionally, these results indicated that, even if nutritional status at enrollment is within the normal range, the nutritional status of COPD patients with changes in emphysematous progression deteriorates over time. An imbalance between protein synthesis and myogenesis has been proposed to underlie muscle wasting in COPD patients^38^, and nutritional supplementation promotes weight gain among COPD patients, especially if they are malnourished^39^. Patients who have related factors of changes in emphysematous progression might be requiring nutritional supplementation and targeted pharmacological interventions.

Recently, several studies have shown that the blood eosinophil count is predictive of exacerbations^40^ and a good response to inhaled corticosteroids^41,42^. Interestingly, even if they are within the normal range, the blood eosinophil count was significantly higher in the non-progression group than in the rapid progression group in the present study. This result is in line with previous studies showing that high eosinophil counts were related to less emphysema^43^, better survival^22,44^, and a slower annual FEV_1_ decline^23^. The specific cause and effect relationship between emphysema progression and low blood eosinophilia is unclear. Previous reports have demonstrated that T helper 1 and 17 cells are relatively abundant in lungs of patients with emphysema compared with those in lungs of former smokers without emphysema^12^. T helper 1-predominant inflammation appears to progress emphysema more rapidly compared to T helper 2-predominant inflammation, a difference that would be related to blood eosinophilia^45^. Blood eosinophil count is thus a simple and inexpensive biomarker predictive of future changes in emphysematous progression.

In COPD patients, the relative contributions of emphysema and small airway disease differ among patients^3,46^. Emphysema-predominance is reported to be associated with greater exercise limitation, reduced QOL^47^, and reduced mortality^48^.

Large clinical trials of COPD patients have shown that current pharmacological treatments have improved lung function^49,50^. Furthermore, Tanabe et al. reported the tiotropium-induced reduction of emphysema volume based on CT images^9^. However, the prognostic value thereof and appropriate therapy for progressive emphysema are unknown. These matters should be considered in future research.

Recent advance of CT metrics has improved phenotyping of COPD. For instance, parametric response mapping identified the extent of functional small airway disease and emphysema^51^. In addition, CT-derived pectoralis muscle area provides a relevant index of COPD morbidity^52^. Further studies that evaluate the relationship among changes in emphysematous progression and these new CT metrics are required.

This study has several strengths. First, the comprehensive assessment of comorbid factors in the K-CCR cohort study^18,19^. Second, assessment of changes in emphysematous progression was based on annual CT over 3 years. In this study, the ΔLAA%/year and not ΔTLV/year correlated with LAA volume. These results imply that the increase in ΔLAA%/year was due to the increase in ΔLAA volume/year, but not that of ΔTLV/year. Emphysema quantification is very sensitive to various conditions, including the level of inspiration, and this issue becomes more important when assessing longitudinal changes by chest CT^13^. Thus, we first carefully performed calibration using a lung phantom and annual CT. The distribution of ΔLAA% is diverse across the previous reports^10,53,54^. In this study, ΔLAA% was normally distributed and about 58.1% of participants were categorized in − 1 to 1 (ΔLAA%) / year. These results were consistent with the previous report^53^, but inconsistent with other report^10^. This discrepancy may be caused by differences in the inspiration levels or different machines.

There were several limitations to this study. First, Japanese COPD patients are reported to have a lower BMI and fewer exacerbations than COPD patients in other countries^29,55^. Thus, this study’s population may not reflect the general COPD population worldwide. Second, the number of females in this study was relatively small. It has been reported that male smokers are more likely to develop emphysema than female smokers^56^. Thus, the findings of our study may not be extrapolatable to female COPD patients. Third, we could not analyze the long-term follow-up outcome such as the rate of hospitalization or mortality. To date, the relationship between changes in of emphysematous progression and these outcomes are unknown. Further studies involving larger number of nested patients and longer follow-up are necessary. Fourth, we could not perform CT using a single CT scanner in this study. Although the calibration among four CT scanners was performed, the differences of CT values in the different scanners might have affected the results.

## Conclusion

The rapid emphysema progression group exhibited a lower eosinophil count, and more often had osteoporosis than the non-progression group. Additionally, rapid progression of emphysema is associated with on-going deterioration of nutritional status in COPD patients. Future studies should focus on appropriate intervention for rapid changes in emphysematous progression and patients who are at risk of rapid emphysematous progression and might be requiring nutritional supplementation and targeted pharmacological interventions.

## Supplementary Information

Supplementary Figures.

Supplementary Tables.

## Data Availability

The data that support the findings of this study are available from the corresponding author upon reasonable request.

## Abbreviations

LAA: Low attenuation area
ΔLAA%/year: Annual changes in LAA%
BMI: Body mass index
BMD: Bone mineral density
COPD: Chronic obstructive pulmonary disease
CI: Confidence interval
CT: Computed tomography
CAT: COPD assessment test
CRP: C-reactive protein
DXA: Dual X-ray absorptiometry
FFM: Fat-free mass
FFMI: FFM index
FEV_1_: Forced expiratory volume in 1 s
%FEV_1_: Forced expiratory volume in 1 s as a percentage of predicted forced expiratory volume in 1 s
GOLD: Global Initiative for Chronic Obstructive Lung Disease
ICS: Inhaled corticosteroids
K-CCR: Keio COPD Comorbidity Research
LAA%: Low attenuation area percentage
LVRS: Lung volume reduction surgery
MM: Muscle mass
OR: Odds ratio
QOL: Quality of life
SAA: Serum amyloid A
sRAGE: Soluble receptor for advanced glycation end products
SGRQ: St. George’s Respiratory Questionnaire
SP-D: Surfactant protein D (SP-D)
TLV: ΔTotal lung volume
WA%: The percentage of airway wall area
