# Supplementary material for: Characteristics of chronic obstructive pulmonary disease patients with robust progression of emphysematous change
Source: Sci Rep. 2021 May 5;11:9548. doi: 10.1038/s41598-021-87724-8 (PMC8099884; doi:10.1038/s41598-021-87724-8)
Supplement: Supplementary file 1 — Supplementary Figures. [file 41598_2021_87724_MOESM1_ESM.pdf]

Supplementary Figures

Supplemental Figure 1. Process of patient selection.

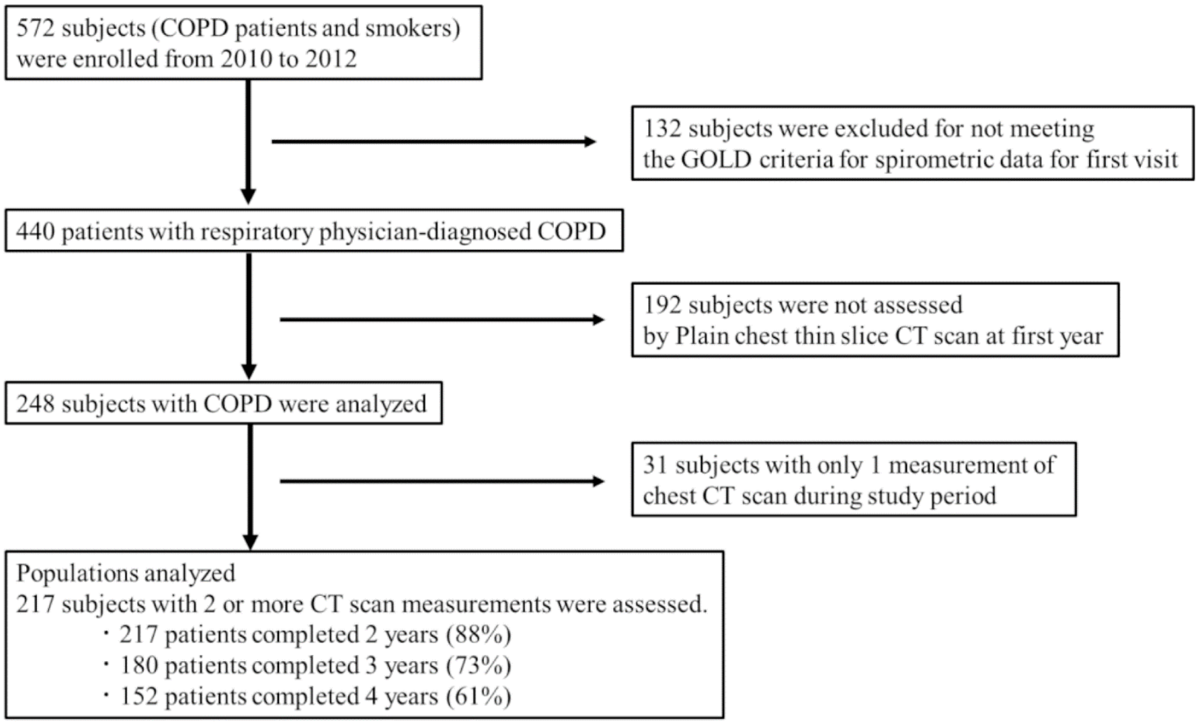

Data of COPD patients with spirometrically confirmed who underwent CT at least two times over a 3-year period (n = 217) were analyzed.

Supplemental Figure 2. Method of calibration of the four computed tomography (CT) scanners.

A)

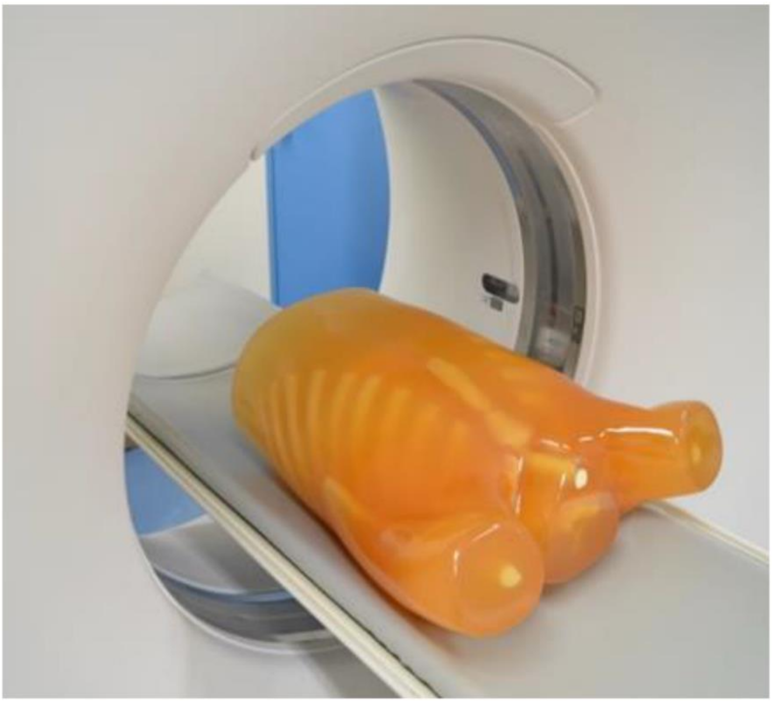

B)

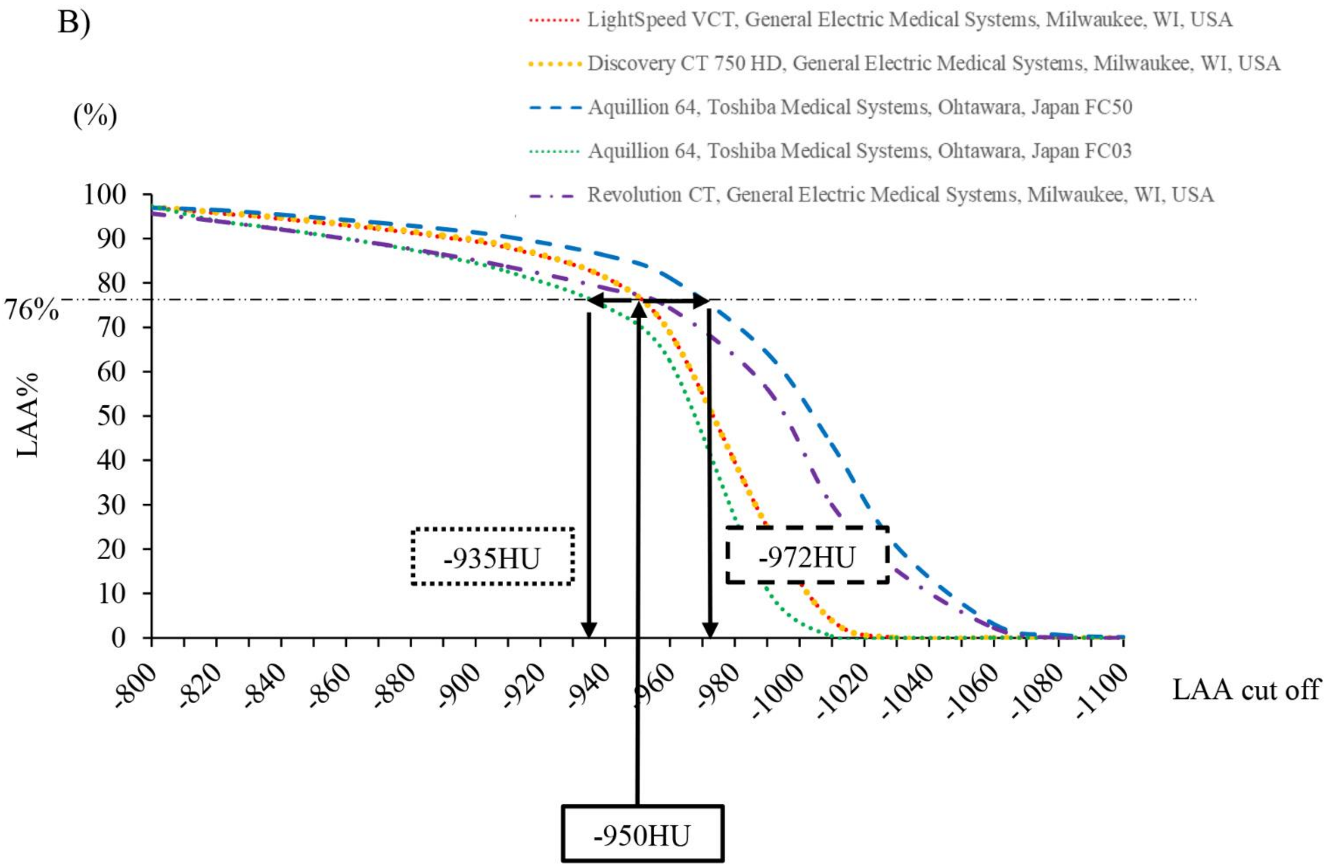

As shown in Supplemental Figure 2A below, the phantom was first scanned on one control CT scanner. The LAA% of this phantom varies depending on the cutoff HU value. When the cutoff LAA value was set at -950 HU on the control CT scanner, LAA% was 76%. The same phantom was scanned on the other four scanners, and the cutoff HU level specific to each model by which LAA% became 76% was determined to allow adjustment (Supplemental Figure 2B).

Supplemental Figure 3. Relationships between femur BMD and changes in emphysematous progression in COPD patients.

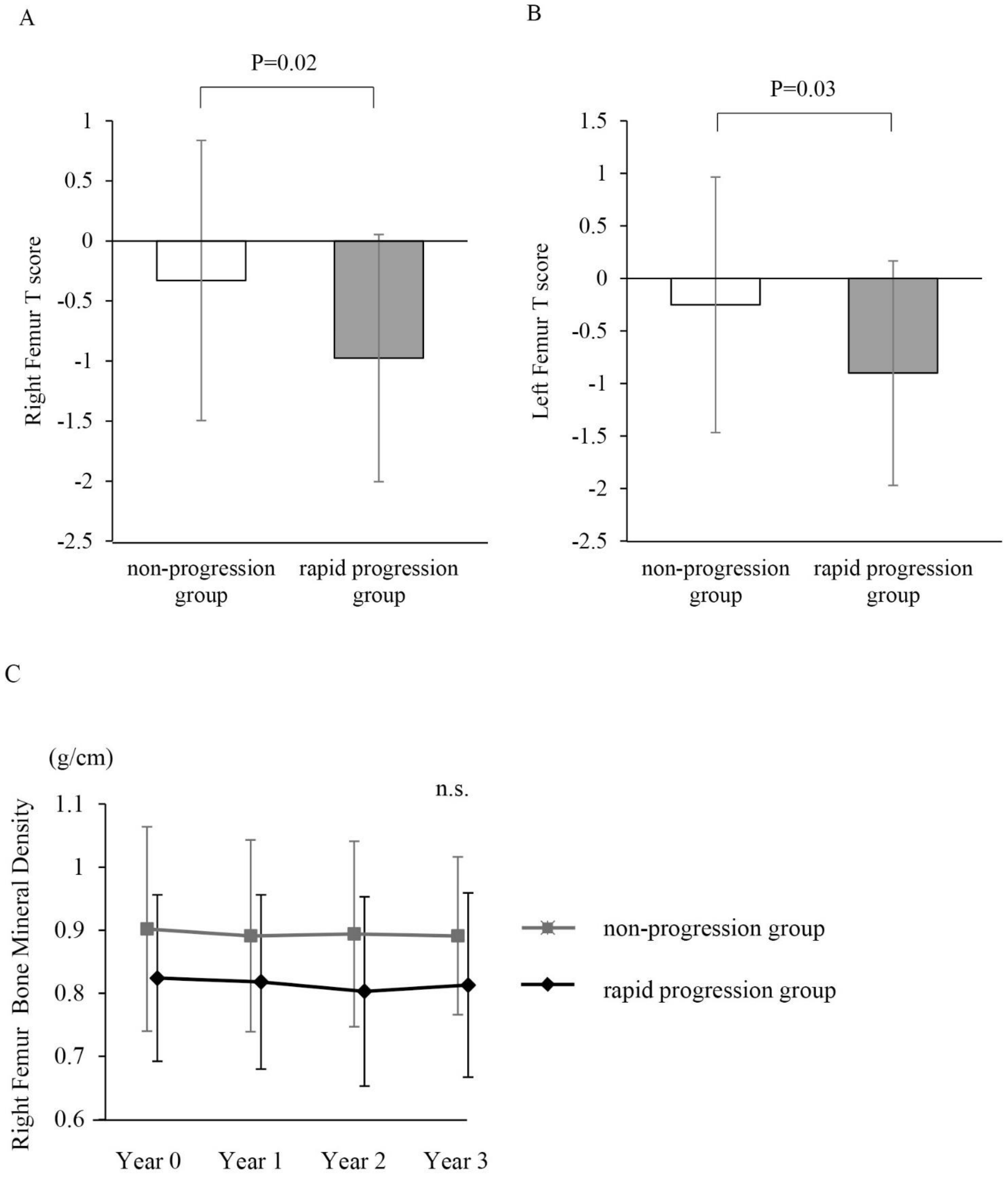

D

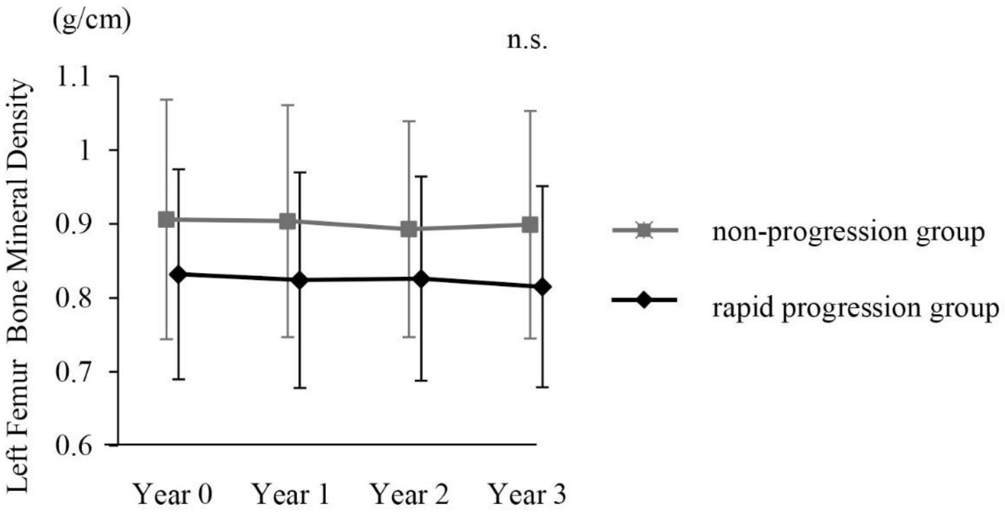

A) Comparison of baseline T score in the right femur between the rapid and non-progression groups.

B) Comparison of baseline T score in the left femur between the two groups.

C) Annual change in BMD in the right femur between the two groups over 3 years of follow-up.

D) Annual change in BMD in the left femur between the two groups over 3 years of follow-up.

Data are shown as mean ± SD. BMD, bone mineral density.

Supplemental Figure 4. Comparison of annual change in health status between the rapid progression group and non-progression group.

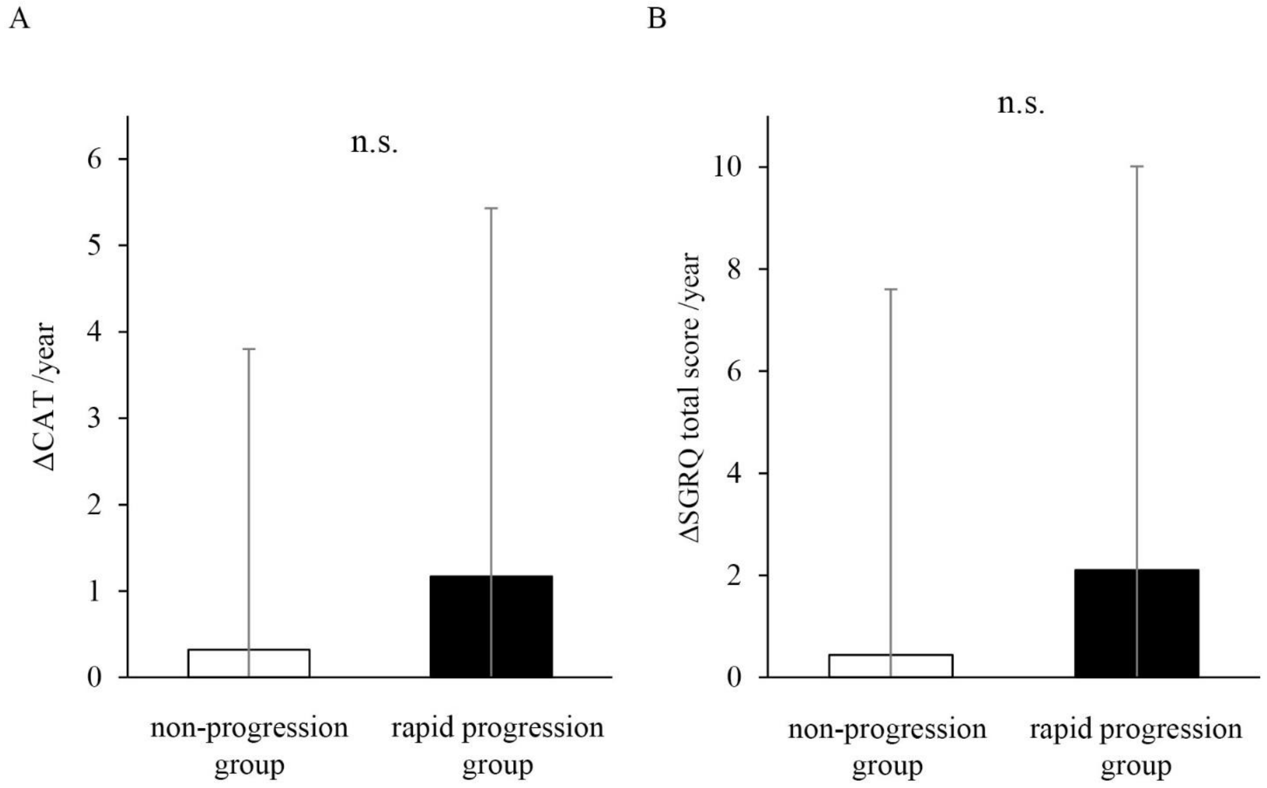

A) Comparison of ΔCAT between the two groups.

B) Comparison of ΔSGRQ between the two groups.

Data are shown as mean ± SD. CAT, COPD assessment test. SGRQ, St. George's Respiratory Questionnaire.
