# Supplementary material for: Characteristics of chronic obstructive pulmonary disease patients with robust progression of emphysematous change
Source: Sci Rep. 2021 May 5;11:9548. doi: 10.1038/s41598-021-87724-8 (PMC8099884; doi:10.1038/s41598-021-87724-8)
Supplement: Supplementary file 2 — Supplementary Tables. [file 41598_2021_87724_MOESM2_ESM.pdf]

Supplementary Tables

Supplemental Table 1 The baseline characteristics of COPD patients stratified by the baseline LAA%

	LAA% < 10% *N = 104*	LAA% 10–20% *N = 55*	LAA% > 20% *N = 58*	P-value
Age, year	72.2 ± 9.2	72.3 ± 7.5	72.7 ± 7.9	0.93
Sex, female, N (%)	9 (8.7)	5 (9.1)	5 (8.6)	0.99
Smoking index, pack-years	48.4 ± 33.3	57.8 ± 27.7	58.4 ± 29.0	0.08
Current smoker, N (%)	14 (13.6)	2 (3.7)	3 (5.4)	0.07
Lung function				
FEV_1, ml	1998.6 ± 544.8	1852.4 ± 641.8	1422.6 ± 601.5	< 0.01
$\%FEV_1$, %	74.4 ± 17.1	69.8 ± 21.4	54.2 ± 21.0	< 0.01
D_{LCO}, mmol/min/mm Hg	14.5 ± 4.3	11.5 ± 4.2	8.9 ± 4.4	< 0.01
$\%D_{LCO}$, %	87.6 ± 21.0	73.8 ± 22.9	60.3 ± 23.6	< 0.01
LAA%, %	4.9 ± 2.8	14.1 ± 2.8	28.2 ± 5.9	< 0.01
Comorbidities				
Interstitial pneumonia, N (%)	7 (6.7)	11 (20.0)	8 (13.8)	0.04
Asthma, N (%)	20 (19.6)	12 (22.2)	11 (19.6)	0.92

Laboratory values				
Blood neutrophil count, cells/mm^3	3777.9 ± 1555.7	3735.1± 1231.4	4298.4 ± 1657.7	0.08
Blood eosinophil count, cells/mm^3	210.8 ± 215.4	225.8 ± 170.8	183.3 ± 174.4	0.50
Patient-reported outcomes				
CAT score	10.5±7.0	12.1±8.3	14.2±8.7	0.02
SGRQ total score	22.4±15.9	27.1±17.4	33.6±20.2	< 0.01
Body Mass Index, (kg/m^2)	24.2±3.1	22.8±2.74	21.2±2.57	< 0.01
Fat Free Mass Index, (kg/m^2)	18.7±2.0	17.3±1.7	16.7±1.8	< 0.01
T-score	-0.71±1.33	-0.86±1.27	-1.39±1.17	< 0.01
Osteoporosis/Osteopenia/ Normal, N (%)	8/32/58 (8.2/32.6/59.2)	6/17/30 (11.3/32.1/56.6)	13/21/20 (24.1/38.9/37.0)	0.03
Bronchodilator (%)	58 (55.8)	38 (69.1)	52 (89.7)	0.01
ICS, N %	18 (17.3)	16 (29.1)	21 (36.2)	0.02

Data are presented as mean ± SD or number (%). LAA%, the ratio of low attenuation area to total lung volume; FEV_1, forced expiratory volume in 1 second; $\%FEV_1$, forced expiratory volume in 1 second as a percentage of predicted forced expiratory volume in

1 second; D_{LCO}, diffusing capacity of the lung for carbon monoxide; % D_{LCO}, diffusing capacity of the lung for carbon monoxide as a percentage of predicted; CAT, chronic obstructive pulmonary disease assessment test, SGRQ, St. George's Respiratory Questionnaire

Supplemental Table 2 Comparison of exacerbation rate and the change of smoking habit according to group

	Non-progression group (*N = 163*)	Rapid progression group (*N = 54*)	P-value
Exacerbation, N (%)	40 (30.5)	9 (27.2)	0.71
Quitting smoking, N (%)	15 (9.2)	4 (7.4)	0.67
New prescription of bronchodilators, N (%)	11 (6.8)	2 (3.7)	0.41
New prescription of ICS, N (%)	22 (13.5)	6 (11.1)	0.65
